# Prenatal Inflammation-Induced Hypoferremia Alters Dopamine Function in the Adult Offspring in Rat: Relevance for Schizophrenia

**DOI:** 10.1371/journal.pone.0010967

**Published:** 2010-06-04

**Authors:** Argel Aguilar-Valles, Cecilia Flores, Giamal N. Luheshi

**Affiliations:** Douglas Mental Health University Institute, Department of Psychiatry, McGill University, Montreal, Quebec, Canada; Singapore Immunology Network, Singapore

## Abstract

Maternal infection during pregnancy has been associated with increased incidence of schizophrenia in the adult offspring. Mechanistically, this has been partially attributed to neurodevelopmental disruption of the dopamine neurons, as a consequence of exacerbated maternal immunity. In the present study we sought to target hypoferremia, a cytokine-induced reduction of serum non-heme iron, which is common to all types of infections. Adequate iron supply to the fetus is fundamental for the development of the mesencephalic dopamine neurons and disruption of this following maternal infection can affect the offspring's dopamine function. Using a rat model of localized injury induced by turpentine, which triggers the innate immune response and inflammation, we investigated the effects of maternal iron supplementation on the offspring's dopamine function by assessing behavioral responses to acute and repeated administration of the dopamine indirect agonist, amphetamine. In addition we measured protein levels of tyrosine hydroxylase, and tissue levels of dopamine and its metabolites, in ventral tegmental area, susbtantia nigra, nucleus accumbens, dorsal striatum and medial prefrontal cortex. Offspring of turpentine-treated mothers exhibited greater responses to a single amphetamine injection and enhanced behavioral sensitization following repeated exposure to this drug, when compared to control offspring. These behavioral changes were accompanied by increased baseline levels of tyrosine hydroxylase, dopamine and its metabolites, selectively in the nucleus accumbens. Both, the behavioral and neurochemical changes were prevented by maternal iron supplementation. Localized prenatal inflammation induced a deregulation in iron homeostasis, which resulted in fundamental alterations in dopamine function and behavioral alterations in the adult offspring. These changes are characteristic of schizophrenia symptoms in humans.

## Introduction

Environmental factors, combined with genetic predisposition, are now recognized as key events underlying a number of psychiatric disorders of neurodevelopmental origin, including schizophrenia. Of the environmental events, maternal infection during critical stages of human gestation has been associated with increased incidence of schizophrenia in the adult progeny [1]–[4]. Studies in animal models of maternal infection have demonstrated a plethora of behavioral, molecular and structural alterations relevant to schizophrenia in the adult offspring [5]–[16]. The mechanisms responsible for these alterations are unknown. However, because a wide variety of viral and bacterial pathogens are implicated [2], [17], it is thought that a response common to all forms of infection is involved in the etiology of the disorder [1], [18]. One such response is hypoferremia, a cytokine-mediated reduction of circulating non-heme iron [19]–[22]. In normal individuals, this response is triggered to limit the availability of this essential nutrient to the invading pathogens and is thus considered an inherent protective mechanism [23]–[25]. Hypoferremia results from the hepcidin (HAMP)-mediated interruption of iron trafficking into the blood from body stores such as macrophages, hepatocytes and from duodenal enterocytes, which mediate dietary absorption of iron [25], [26]. Hypoferremia during pregnancy may have serious repercussions for the developing fetus. Sufficient iron supply is necessary for neurodevelopmental processes; in fact, reduction in iron supply at several stages of development results in enduring changes in dopamine (DA) neurotransmission [27]–[30] that outlast the iron deficient periods [28], [31].

We hypothesized that inflammation-induced hypoferremia causes a disruption of fetal brain development, which leads to functional defects in adulthood, synonymous with psychiatric disorders such as schizophrenia. The goal of this study was, therefore, to investigate whether changes in iron traffic induced by maternal inflammation would have an impact on DA function and DA-related behaviors in the adult offspring. To this end, we conducted studies using a rat model of localized injury and inflammation induced by an intramuscular (i.m.) injection of turpentine (TURP). In contrast to the more commonly used models of systemic bacterial [lipopolysaccharide (LPS)] or viral (poly I:C) infection, TURP remains localized at the injection site [32]. This feature allows us to study the role of endogenous inflammatory mediators on fetal development, in the absence of possible confounding factors triggered by systemically injected immunogens, which could act directly on the fetal compartment [33]–[37] and often lead to high maternal mortality [7]. In recent studies, we demonstrated that treatment of pregnant rats with TURP induces reproducible behavioral changes in the adult offspring, similar to those induced by maternal LPS or poly I:C treatments [7]. The aim of the present study was to investigate whether these effects are due to alteration in DA function resulting from reduced iron levels during a critical neurodevelopmental period (i.e. gestational day [GD] 15). Our results strongly support a major role for inflammation-induced hypoferremia in the development of enhanced DA function in the offspring.

## Results

### TURP induces an acute-phase response and hypoferremia in GD 15 pregnant dams

**Figure 1:**
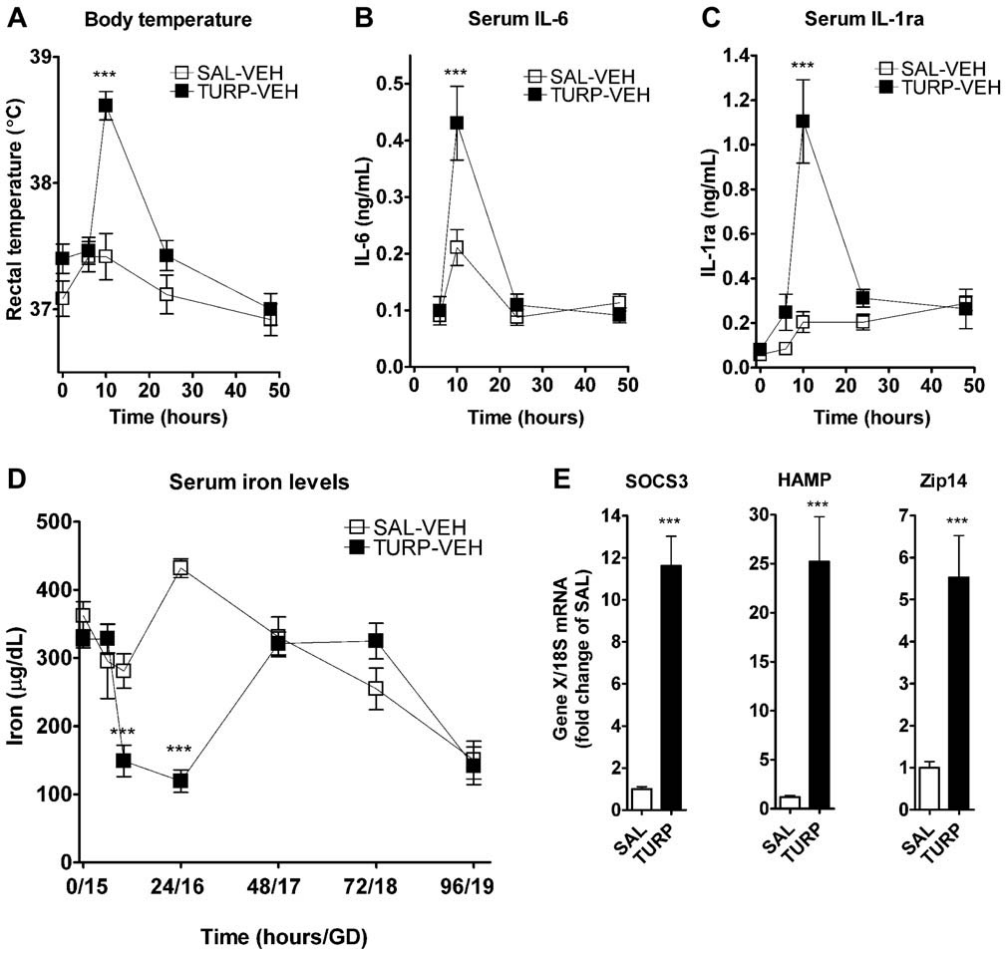
TURP induced a maternal inflammatory response and hypoferremia in pregnant rats at GD 15. (A) Sterile saline (n = 6) or TURP (n = 8) was injected (i.m., 100 µl/rat) and core body temperature determined immediately before (0 time point) and 8, 10, 24 and 48 h after. TURP induced a significant increase in core body temperature 10 h after TURP injection, which receded 24 h after injection. *p*<0.0001: *** SAL vs. TURP (B and C) Serum IL-6 and IL-1ra levels were determined by ELISA from samples collected 6, 10, 24 and 48 h after SAL or TURP injections in the same rats as in (A). TURP induced a significant increased in the levels of both cytokines 10 h after injection and returned to baseline by 24 h. *p*<0.0001: *** SAL vs. TURP (D) Non-heme iron levels were also measured in the serum samples from the same animals used in (A-C). TURP induced significant reduction in non-heme levels 10 and 24 hours after TURP injection, returning to control levels by 48 hours. Non-heme iron levels decreased as pregnancy progressed, independently of treatment. *p*<0.0001: *** SAL vs. TURP (E) Hepatic gene expression in dams sacrificed at the peak of fever and cytokine induction, 10–11 hours after i.m. injection. SOCS3 (left panel), HAMP (middle panel) and zip14 (right panel) mRNAs were significantly induced in the TURP treated dams (n = 7 per group). *p*<0.0001: *** SAL vs. TURP.

In order to characterize the maternal inflammatory response to TURP and the alterations that this may induce in systemic iron trafficking, we determined the kinetics of several inflammatory markers and maternal serum iron levels at GD 15. TURP induced a significant increase in core body temperature at 10 hours, with a 1.9°C difference in comparison to the saline (SAL) group and returned to basal 24 h after injection (Fig. 1A; treatment by time interaction, F_(4, 48)_ = 7.74, *p*<0.001, simple effect of treatment at 10 h: F_(1, 53.84)_ = 37.82, *p*<0.0001). Serum pro- [interleukin (IL)-6)] and anti- [IL-1 receptor antagonist (IL-1ra)] inflammatory cytokines were significantly induced at the same time point (10 h) after TURP injection and no longer detectable by 24 h (GD 16; Fig. 1B and C, respectively; IL-6: treatment by time interaction, F_(5, 60)_ = 3.98, *p* = 0.004, simple effect of treatment at 10 h F_(1, 69.28)_ = 17.5, *p*<0.0001; IL-1ra: treatment by time interaction F_(6, 62)_ = 11.04, *p*<0.0001, simple effect of treatment at 10 h F_(1, 71.39)_ = 52.2, *p*<0.0001).

TURP treatment dramatically reduced serum non-heme iron levels, 10 and 24 h after injection (Fig. 1D; treatment by time interaction, F_(6, 72)_ = 14.10, *p*<0.0001; simple effect of treatment at 10 h, F_(1, 78.88)_ = 12.34, *p* = 0.0007; at 24 h, F_(1,78.88)_ = 69.26, *p*<0.0001), returning to control levels at 48 h (GD 17) after treatment. No further differences in non-heme iron levels were observed between TURP and control groups. However, there was a reduction in the serum levels of iron with the progress of pregnancy towards term, with non-heme iron levels at GD 19 becoming significantly lower than those at GD 15, 16, 17 and 18 (Fig. 1D; main effect of time, F_(6, 72)_ = 16.36, *p*<0.0001, 96 h vs. 0, 24, 48 or 72 h Fisher's LSDs *p*< 0.05). This decrease in maternal serum non-heme iron towards term is in fact normally observed, as iron is used to sustain increased requirement for this nutrient due to expansion of the maternal erythrocyte mass and the high demand of the growing fetus [38], [39].

TURP's effect on circulating iron levels was accompanied by an induction of hepatic expression levels of HAMP mRNA by 25-fold (Fig. 1E; t_(12)_ = 5.27, *p* = 0.0002) and zip14 mRNA by 5.5-fold (Fig. 1E; t_(12)_ = 4.48, *p* = 0.0007). Zip14 is a dual iron/zinc importer involved in the cellular uptake of iron [40]. In addition, we observed an 11.6-fold increase in the expression levels of suppressor of cytokine signaling (SOCS) 3 mRNA after TURP treatment (Fig. 1E; t_(12)_ = 7.6, *p*<0.0001), indicating the activation of the JAK/STAT signaling pathway, most likely by IL-6 [41], which is involved in the induction of HAMP and zip14 mRNA expression.

### Maternal iron supplementation reverses hypoferremia, but has no effect on other inflammatory responses

**Figure 2:**
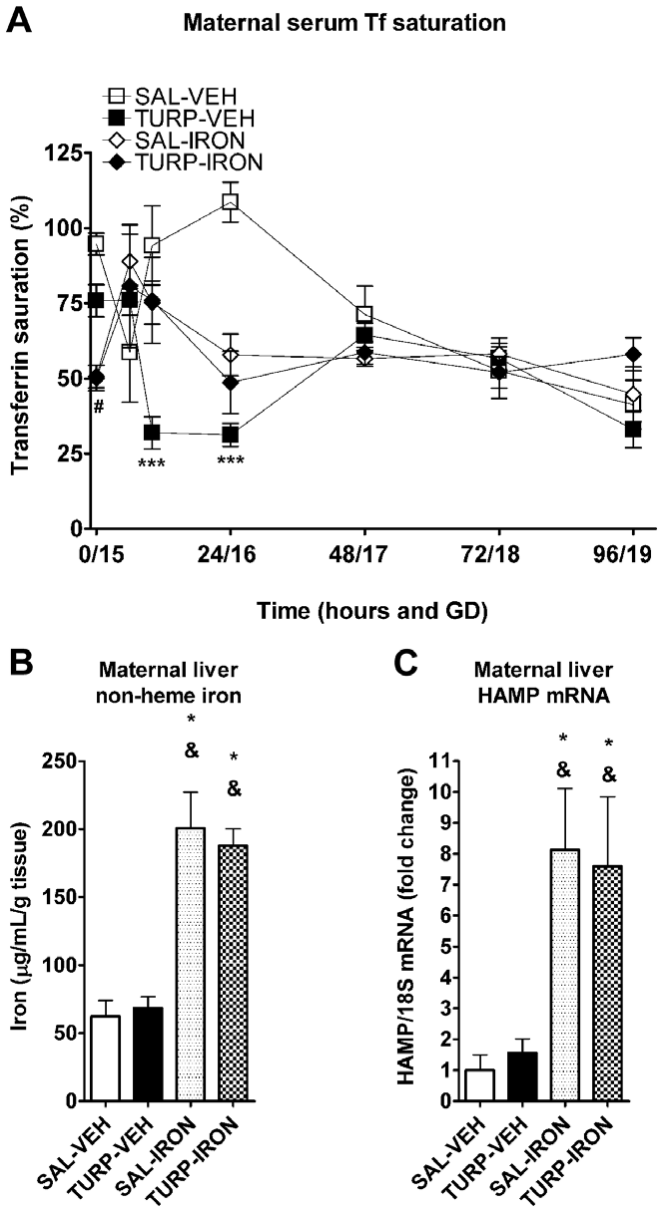
TURP effect on maternal serum Tf saturation was prevented by parenteral iron supplementation. (A) Serum Tf saturation levels were significantly reduced 10 and 24 h after TURP injection, this effect was absent in TURP-IRON treated dams. Baseline Tf saturation levels in iron supplemented animals was significantly reduced. (B and C) Hepatic non-heme iron levels, 96 h after i.m. injection, were unaffected by TURP treatment, but dramatically increased in iron supplemented dams. In parallel, iron supplementation led to a strong induction maternal hepatic HAMP mRNA expression. *p*<0.05 * vs. SAL-VEH, & vs. TURP-VEH.

We determined if parenteral iron supplementation had any side effect on the inflammatory responses induced by TURP, namely cytokine production or fever. Maternal febrile response, as well as induction of IL-6 and IL-1ra by TURP remained intact following the iron supplementation schedule, with no significant effects of iron supplementation detectable on all three parameters (Fig. S1A-C in Supporting Information). Maternal serum non-heme iron levels, analyzed as transferrin (Tf) saturation, were differentially affected by TURP, depending on the maternal supplementation (Fig. 2A). Tf saturation was strongly diminished by TURP treatment in vehicle (VEH) co-treated animals (Fig. 2A; prenatal treatment by maternal supplementation interaction, F_(1, 23)_ = 7.85, *p* = 0.01; simple effect of maternal treatment for VEH supplemented animals, F_(1, 23)_ = 24.40, *p*<0.0001), supporting the effect seen by analyzing raw serum non-heme iron levels (Fig. 1D). Importantly, TURP treatment did not have any effect on serum Tf saturation in the animals supplemented with iron (Fig. 2A; simple effect of maternal treatment for iron supplemented animals, F_(1, 23)_ = 0.95, *p* = 0.33), suggesting a blockade of the TURP effect on serum iron levels when mothers were over-loaded with this micronutrient. Basal Tf saturation among the iron supplemented animals was significantly lower than VEH mothers (supplementation by time interaction, F_(5, 115)_ = 4.91, *p* = 0.0004, simple effect of supplementation at 0 h, F_(1, 135.6)_ = 10.8, *p* = 0.0013), which may be due to the induction of hepatic HAMP mRNA expression, as observed in both SAL-IRON and TURP-IRON groups at GD 19 (96 h after i.m. injections; Fig. 2C; effect of treatment, F_(3, 21)_ = 5.89, *p* = 0.044; SAL-IRON or TURP-IRON vs. SAL-VEH or TURP VEH Fisher's LSDs, *p*<0.05). Accordingly, there was an accumulation of non-heme iron in the maternal liver of iron-supplemented dams at this same time point (Fig. 2B; effect of treatment, F_(3, 22)_ = 23.18, *p*<0.0001; SAL-IRON or TURP-IRON vs. SAL-VEH or TURP VEH Fisher's LSDs, *p*<0.0001), in the absence of all the other TURP-induced effects, which returned to basal/control levels [maternal serum non-heme iron (Fig. 1D); hepatic non-heme iron content (Fig. 2B) and maternal hepatic HAMP mRNA expression (Fig. 2C)].

### TURP induces long-term effects on fetal iron content, which are prevented by maternal iron supplementation

**Figure 3:**
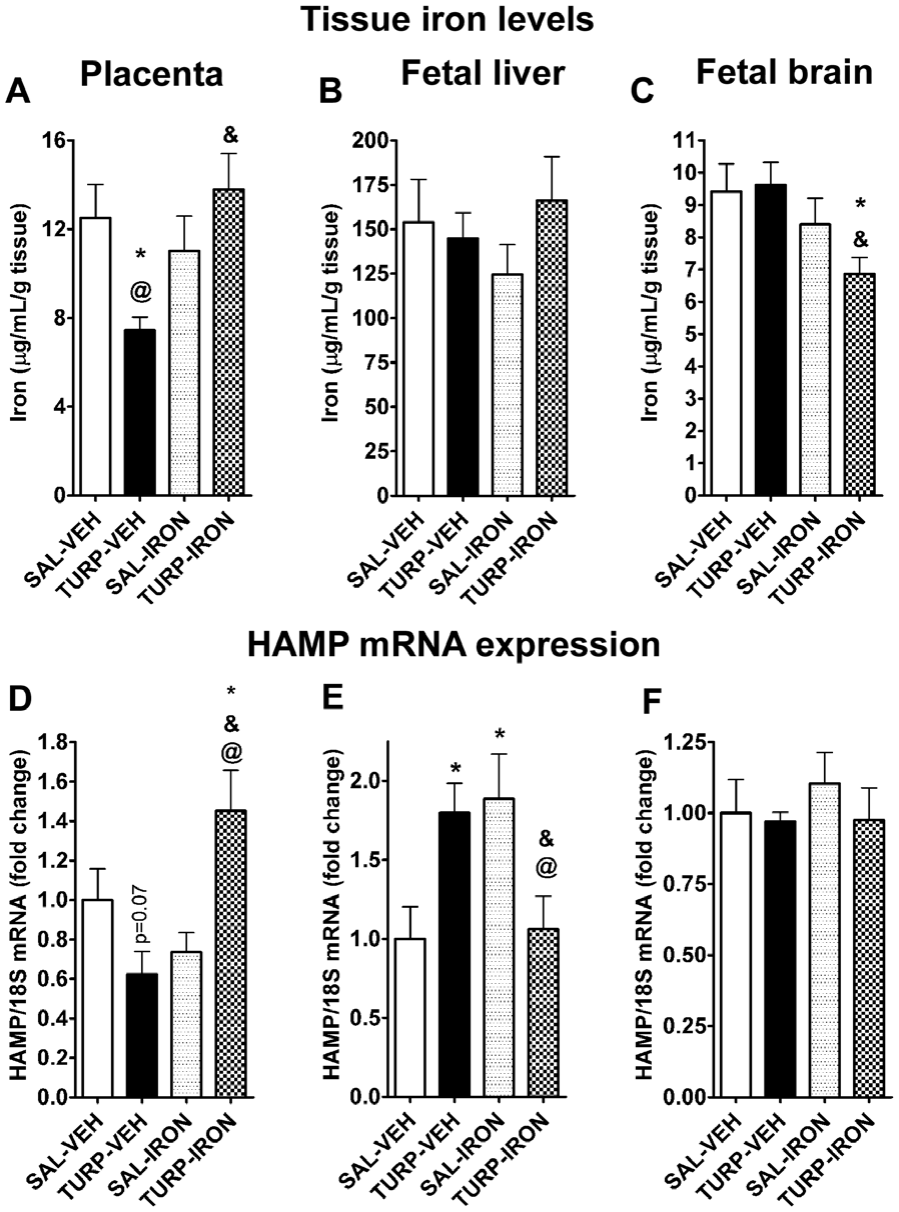
Fetal non-heme iron and HAMP mRNA levels are modulated by maternal TURP treatment and iron supplementation. (A) Non-heme iron levels in the placenta were lower in the TURP-treated dams, and this effect was reversed in the TURP-IRON treated dams. *p*<0.05: * vs. SAL-VEH, & vs. TURP-VEH, @ vs. SAL-IRON. SAL-VEH n = 6, TURP-VEH n = 8, SAL-IRON n = 7 and TURP-IRON n = 6. (B) Fetal liver non-heme iron content was unaffected by maternal treatments. (C) In the fetal brain, the only significant effect was a significant decrease in the non-heme iron content of TURP-IRON treated mothers. *p*<0.05: * vs. SAL-VEH, & vs. TURP-VEH. (D) Placental HAMP mRNA expression was decreased in TURP-VEH mothers, whereas this effect was significantly reversed in the TURP-IRON treated dams. *p*<0.05: * vs. SAL-VEH, & vs. TURP-VEH, @ vs. SAL-IRON. (E) In the fetal liver, HAMP mRNA expression was significantly increased by TURP and by iron supplementation alone, whereas when both treatments were given together these effects is blocked. *p*<0.05: * vs. SAL-VEH, & vs. TURP-VEH, @ vs. SAL-IRON. (F) HAMP mRNA expression was not significantly altered by any treatment in the fetal brain.

We performed fetal iron content and HAMP mRNA expression measurements 96 h after TURP treatment. This time point was chosen since the acute inflammatory responses to TURP (i.e. maternal fever, cytokine induction and decreased serum non-heme iron) had returned to basal/control levels, enabling us to study the extended effects of the inflammatory challenge on fetal tissue in the absence of the maternal immune responses. In the placenta, non-heme iron content was significantly reduced in the TURP-VEH treated group (effect of treatment, F_(3, 71)_ = 4.36, *p* = 0.007; SAL-VEH vs. TURP-VEH Fisher's LSD, *p*<0.05), and this effect was reversed by iron supplementation in TURP-IRON treated dams (Fig. 3A; TURP-VEH vs. TURP-IRON Fisher's LSD, *p*<0.05). HAMP mRNA expression levels in this organ paralleled the effects seen in iron content, with a reduction induced by TURP treatment (effect of treatment, F_(3, 22)_ = 6.37, *p* = 0.0029; non significant SAL-VEH vs. TURP-VEH Fisher's LSD, *p* = 0.07) and a recovery of HAMP mRNA expression in the TURP-IRON group, to levels significantly greater than those of SAL-VEH (Fig. 3D; TURP-IRON vs. SAL-VEH or TURP VEH or SAL-IRON Fisher's LSDs, *p*<0.05).

There were no effects on fetal liver iron content (Fig. 3B; effect of treatment, F_(3, 68)_ = 0.71, *p* = 0.55). However, fetal hepatic HAMP mRNA levels were significantly increased in the fetuses from TURP- and SAL-IRON treated mothers (Fig. 3E; effect of treatment, F_(3, 21)_ = 4.17, *p* = 0.018; SAL-VEH vs. TURP-VEH or SAL-IRON Fisher's LSD, *p*<0.05), whereas in fetal livers from TURP-IRON treated mothers, HAMP mRNA levels were back to control levels (Fig. 3E; TURP-IRON vs. SAL-IRON or TURP-VEH Fisher's LSDs, *p*<0.05).

In the fetal brain, non-heme iron content was significantly reduced in the fetuses of TURP-IRON treated dams, compared to the fetal brains from SAL-VEH and TURP-VEH treated mothers (Fig. 3C; effect of treatment, F_(3, 73)_ = 2.96, p = 0.038; TURP-IRON vs. SAL-VEH or TURP-VEH Fisher's LSDs, *p*<0.05). However, there were no effects on HAMP mRNA expression (Fig. 3F; effect of treatment, F_(3, 23)_ = 0.46, *p* = 0.71).

### Maternal inflammation and iron supplementation differentially alter sensitivity to the effects of AMPH on locomotor activity in the adult offspring

We first assessed the effects on litter size and body weight at birth, and found that none of the prenatal treatments had a significant effect on these variables (data not shown), suggesting that neither TURP and/or iron supplementation induced fetal mortality nor gross physical changes at birth.

**Figure 4:**
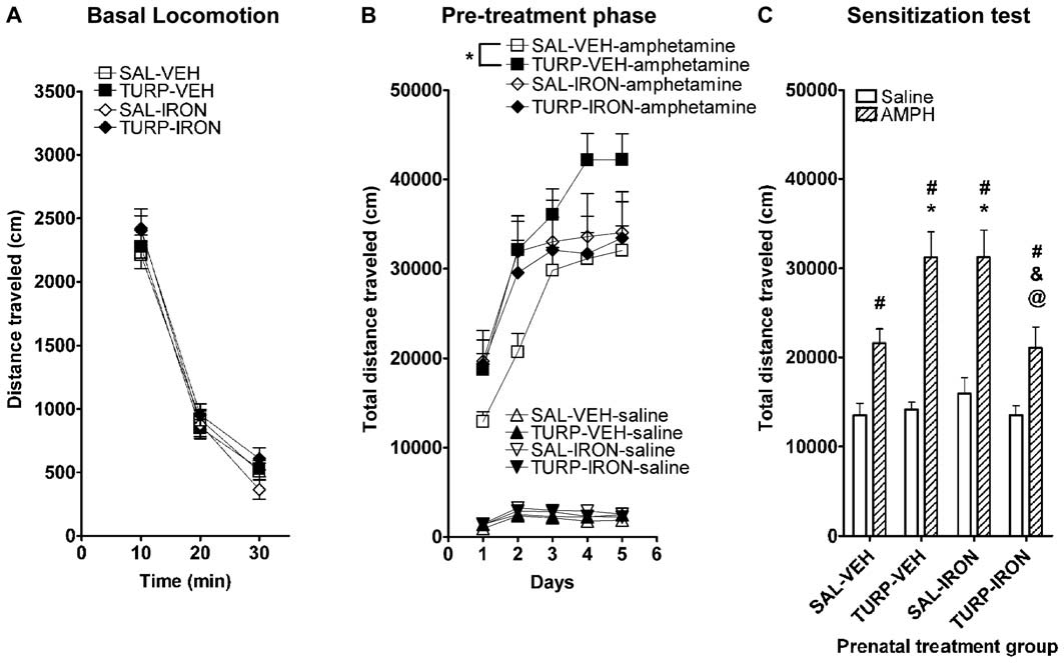
Prenatal TURP treatment increased acute and sensitized AMPH-induced locomotion, which are prevented by maternal iron co-administration. (A) Basal locomotion in the adult offspring of SAL-VEH (n = 45), TURP-VEH (n = 50), SAL-IRON (n = 23) and TURP-IRON (n = 26) treated dams was not affected by any treatment when animals were introduced to the behavioral chambers for the first time. (B) AMPH administration (i.p. 2 mg/kg) for 5 consecutive days induced a progressive enhancement of the locomotor activating effects, which was overall grater among the offspring of TURP-VEH treated dams compare to their SAL-VEH counterparts. This effect was completely prevented by iron co-treated, with the offspring of the TURP-IRON and SAL-IRON groups showing identical levels of locomotor behavior, which were also indistinguishable from those of the SAL-VEH group. Saline pre-treated: SAL-VEH n = 24, TURP-VEH n = 26, SAL-IRON n = 12 and TURP-IRON n = 12. AMPH pre-treated: SAL-VEH n = 21, TURP-VEH n = 24, SAL-IRON n = 11 and TURP-IRON n = 14. (C) Seven days after the last saline or AMPH injection, all animals were tested for sensitization, with a lower dose of AMPH (i.p. 1 mg/kg). All AMPH pre-treated animals showed significantly greater response to this dose of AMPH (dashed bars) than the saline-pretreated animals (white bars). Among the AMPH pre-treated animals, those born to TURP-VEH and SAL-IRON presented significantly greater sensitization response, whereas in the offspring of TURP-IRON dams, sensitization response was comparable to control animals. *p*<0.01: # vs. saline pre-treated counterpart, * vs. AMPH pre-treated SAL-VEH, & vs. AMPH pre-treated TURP-VEH, @ vs. AMPH pre-treated SAL-IRON.

In the adult offspring, locomotion in basal conditions or in response to amphetamine (AMPH, 2 mg/kg of body weight) or saline injection was determined. AMPH or saline were administered to animals of each experimental group for 5 consecutive days. No effects were observed in basal locomotion (Fig. 4A) or in response to injection of saline throughout the 5 days of pre-treatment (Fig. 4B) for individuals born to TURP and SAL-treated mothers, supplemented with either VEH or iron. However, when treated with AMPH, animals born to TURP-VEH mothers showed significantly greater locomotion throughout the 5 pre-treatment days, in comparison to their SAL-VEH counterparts (Fig. 4B; prenatal treatment by supplementation interaction, F_(1, 62)_ = 4.84, *p* = 0.032; simple effect of treatment in the VEH condition, F_(1, 62)_ = 6.96, *p* = 0.01). In contrast, there was no difference in AMPH-induced locomotion between the offspring of TURP and SAL-treated, iron-supplemented mothers (simple effect of treatment in the iron condition, F_(1, 62)_ = 0.22, *p* = 0.64), which exhibited locomotor responses to AMPH statistically similar to those of the SAL-VEH group (Fig. 4B; simple effect of supplementation in the SAL condition, F_(1, 62)_ = 2.32, *p* = 0.13). During days 1 and 2, iron supplemented animals presented a trend for enhanced response to AMPH in comparison to SAL-VEH, which disappeared from day 3 onwards; this effect did not reach statistical significance.

In the test for behavioral sensitization (7 days after last pre-treatment injection), all animals were challenged with a single injection of AMPH (1 mg/kg). As expected, all AMPH pre-treated rats exhibited sensitized response to the AMPH challenge in comparison to those pre-treated with saline (Fig. 4C; main effect of pre-treatment, F_(1, 129)_ = 63.9, *p*<0.0001). However, among the AMPH pre-treated animals, offspring of dams exposed to TURP-VEH showed a significantly greater sensitized response than the offspring of the SAL-VEH group (Fig. 4C; three-way prenatal treatment by maternal supplementation by pre-treatment interaction, F_(1, 129)_ = 7.83, *p* = 0.006; simple effect of prenatal treatment for the AMPH pre-treated VEH animals, F_(1, 129)_ = 10.39, *p* = 0.0016). In contrast, offspring of TURP-IRON treated dams were sensitized to AMPH similar to the SAL-VEH offspring (Fig. 4C; simple effect of maternal supplementation for the AMPH pre-treated TURP animals, F_(1,129)_ = 12.17, *p* = 0.0007). Intriguingly, iron supplementation alone during pregnancy, also resulted in enhanced behavioral sensitization in the adult offspring (Fig. 4C; simple effect of maternal supplementation for the AMPH pre-treated SAL animals, F_(1,129)_ = 9.94, *p* = 0.02; simple effect of treatment for the AMPH pre-treated IRON animals, F_(1, 129)_ = 8.77, *p* = 0.0037).

### Maternal inflammation leads to biochemical alterations in the adult DA system, which are partly reversed by maternal iron supplementation

**Figure 5:**
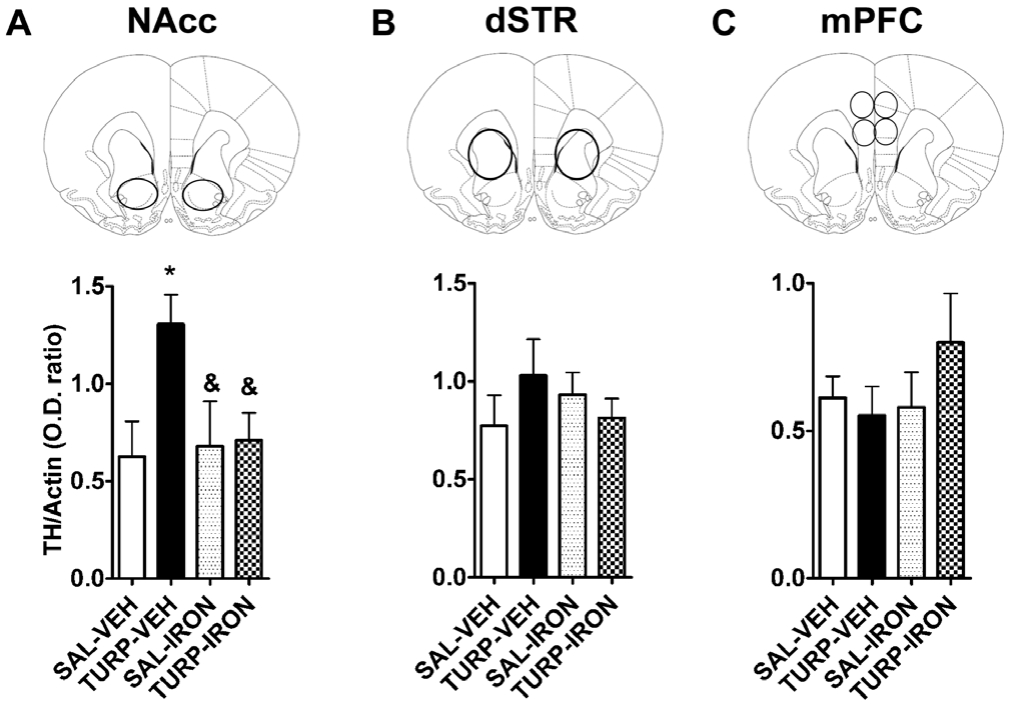
Iron supplementation prevented increased NAcc TH levels induced by prenatal TURP treatment. (A) Basal TH levels in the NAcc were measured by western blotting from adult animals' protein extracts. These were significantly greater in the offspring of TURP-treated dams, which was prevented by maternal iron supplementation. SAL-VEH n = 5, TURP-VEH n = 7, SAL-IRON n = 5 and TURP-IRON n = 6. *p*<0.05 * vs. SAL-VEH, & vs. TURP-VEH. (B and C) TH protein levels from dSTR (B) and mPFC (C) were not significantly affected by any prenatal treatment.

**Figure 6:**
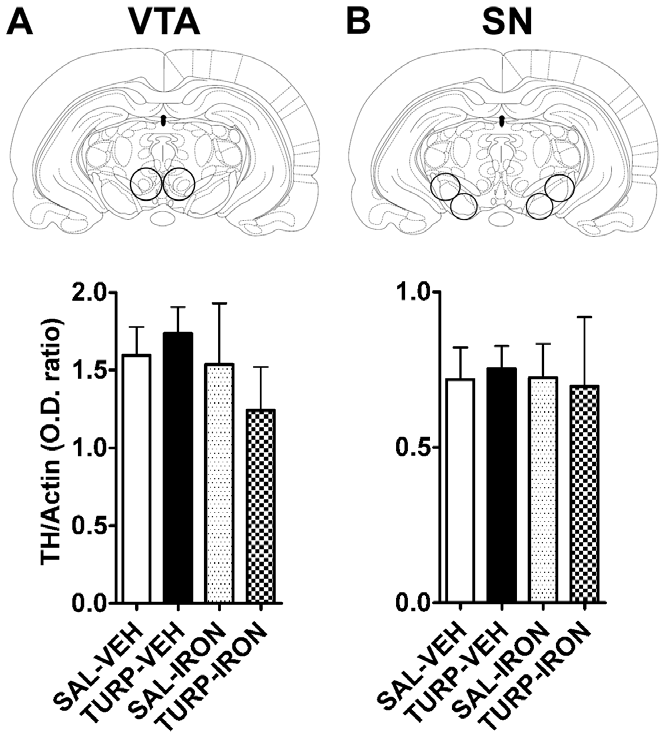
TH expression in the somatodendritic DA regions is not affected by maternal treatments. (A and B) TH protein levels were not significantly altered by maternal manipulations in VTA (A) and SN (B).

We analyzed baseline tyrosine hydroxylase (TH) expression in DA rich regions by Western blotting (Fig. 5). Consistent with our behavioral results, nucleus accumbens (NAcc) TH expression was significantly increased in the adult offspring of the TURP-VEH group compared to the saline controls (Fig. 5A). This effect was not observed in the offspring of iron supplemented mothers (TURP-IRON and SAL-IRON groups), whose levels of TH were comparable to those of the control group (Fig. 5A; effect of treatment, F_(3, 23)_ = 3.63, *p* = 0.028; TURP-VEH vs. SAL-VEH or TURP-IRON Fisher's LSDs, *p*<0.05). These changes were region specific, as levels of TH expression in the dorsal striatum (dSTR; Fig. 5B; effect of treatment, F_(3, 22)_ = 0.62, *p* = 0.60), medial prefrontal cortex (mPFC; Fig. 5C; effect of treatment, F_(3, 21)_ = 0.88, *p* = 0.46), ventral tegmental area (VTA; Fig. 6A; effect of treatment, F_(3, 23)_ = 0.58, *p* = 0.63) and substantia nigra (SN; Fig. 6B; effect of treatment, F_(3, 23)_ = 0.04, *p* = 0.96) were not significantly altered by any prenatal treatment.

**Figure 7:**
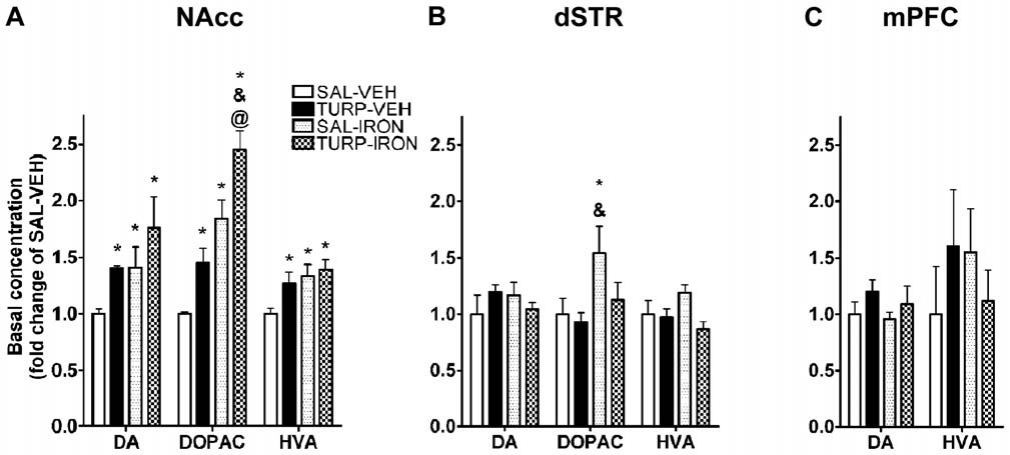
Prenatal inflammation and iron supplementation increase NAcc levels of DA, DOPAC and HVA. (A) In the NAcc prenatal TURP induced a significant induction in the content of DA, DOPAC and HVA. These effects were also present in the offspring of SAL-IRON-treated dams and in the TURP-IRON offspring, who also showed DOPAC levels greater that all of the other groups. SAL-VEH n = 5, TURP-VEH n = 7, SAL-IRON n = 5 and TURP-IRON n = 6. *p*<0.05 * vs. SAL-VEH, & vs. TURP-VEH, @ vs. SAL-IRON. (B) In the dSTR only DOPAC content was significantly greater in the offspring of SAL-IRON dams, whereas no other significant effect was found on the metabolite or in DA and HVA levels. (C) In contrast, in the mPFC no changes were detected in DA and HVA (DOPAC was under detection limits of the assay for this region).

We also measured tissue content of DA, 3,4-dihydroxyphenylacetic acid (DOPAC) and homovanillic acid (HVA) in DA terminal regions (Fig. 7). Consistent with effects seen in TH expression, NAcc levels of DA in the TURP-VEH group were elevated (Fig. 7A; effect of treatment, F_(3, 18)_ = 3.83, *p* = 0.028; SAL-VEH vs. TURP-VEH Fisher's LSD, *p*<0.05), as well as those of DOPAC (effect of treatment, F_(3, 18)_ = 18.27, *p*<0.0001; SAL-VEH vs. TURP-VEH Fisher's LSDs, *p*<0.05) and HVA (effect of treatment, F_(3, 18)_ = 3.83, *p* = 0.041, SAL-VEH vs. TURP-VEH Fisher's LSDs, *p*<0.05). Intriguingly, DA levels in the SAL-IRON and TURP-IRON groups were also increased (Fig. 7A; SAL-VEH vs. SAL-IRON or TURP-IRON Fisher's LSDs, p<0.05); the same was true for NAcc HVA and DOPAC (SAL-VEH vs. SAL-IRON or TURP-IRON Fisher's LSDs, p<0.05), with DOPAC levels in TURP-IRON animals being even greater than the TURP-VEH or SAL-IRON groups (Fig. 7A; TURP-IRON vs. SAL-IRON and TURP-VEH Fisher's LSDs, *p*<0.05). As for TH, effects of prenatal treatments appeared to be specific to the NAcc since neither dSTR (Fig. 7B) nor mPFC (Fig. 7C) DA and HVA were altered and dSTR DOPAC was significantly increased only in the offspring of SAL-IRON treated dams (Fig. 7B; effect of treatment, F_(3, 20)_ = 3.26, *p* = 0.043; SAL-VEH vs. SAL-IRON Fisher's LSD, *p*<0.05).

## Discussion

In the current study we provided the first evidence, to our knowledge, that localized maternal inflammation during gestation renders adult offspring significantly more sensitive to the locomotor activating effects of a single AMPH injection, and to the behavioral plasticity following repeated exposure to this drug. In addition, we demonstrated increased baseline expression of TH and tissue levels of DA, DOPAC and HVA, which were specific to the NAcc. The fact that TURP does not enter the circulation suggests that the effects observed in the offspring in the current study, are most likely due to secondary downstream mediators and not to direct effects on the fetal compartment, as reported for poly I:C [12], [42]–[45] and LPS [6], [14], [15], [33], [46], [47].

We demonstrated that maternal iron supplementation, which counteracts inflammation-induced hypoferremia, effectively prevented the prenatal TURP-induced increased sensitivity to the behavioral effects of single and repeated AMPH administration in the adult offspring. These results suggest that maternal hypoferremia, induced by the TURP injection, causes long-lasting behavioral alterations in the offspring. Remarkably, prenatal TURP-induced increase in NAcc TH expression was also completely abrogated by maternal iron supplementation.

Increased levels of TH and DA, restricted to the NAcc, have also been reported by others in the offspring of mothers treated with poly I:C [42] and LPS [46]. As with the effect of TURP, there is no clear explanation on how these prenatal inflammatory insults, leads to changes in TH, DA and metabolites that are restricted to this brain region. One possibility may be linked to the differential distribution of iron in the brain. Iron is particularly abundant within DA-rich regions of the brain, where it has been shown to have a role in DA metabolism and function [28], [30], [48]. Of these regions, the NAcc and the dSTR are among the most enriched areas of the brain [30], and they are sensitive to systemic variations in iron (i.e. dietary restriction or supplementation), which in turn affect DA neurotransmission. Likewise, iron deficiency during gestation has been shown to affect DA neurotransmission in the whole striatum, including the NAcc [30], [48]–[51]. Interestingly, although less studied, mPFC DA neurotransmission seems largely unaffected by early manipulations in iron [28]. This is supported by our findings which show that the effects of TURP on DA was restricted to the NAcc, and that iron supplementation alone affected DA metabolites only in the NAcc and the dSTR, whereas neither treatment affected the mPFC TH, DA or HVA levels. These results suggest that the anatomical localization of iron may underlie the effects of maternal TURP and/or iron supplementation to modulate striatal DA transmission, as opposed to cortical DA function, although it does not fully explain the differential effect between NAcc and dSTR.

Increased NAcc TH, which is consistent with augmented basal NAcc DA concentration, may result from either a change in expression level per neuronal terminal, or from structural reorganization within the mesolimbic circuitry, the latter resulting in axonal sprouting of NAcc DA terminals. Prenatal inflammation may therefore lead to profound presynaptic alterations in NAcc DA synthesis. However, additional effects on DA receptors, transporter [42] and/or catabolic enzymes cannot be excluded, as baseline levels of DOPAC and HVA were also increased by prenatal TURP. Our findings of increased DA function in the NAcc at baseline are likely to contribute to increased behavioral response to acute AMPH injection and perhaps, to the enhanced AMPH-induced behavioral plasticity following repeated treatment [52], [53]. Intriguingly, altered levels of basal NAcc DA, DOPAC and HVA remained increased in the offspring of TURP-IRON treated mothers, despite normalization of TH expression. This suggests additional alterations in the synthetic and/or catabolic pathways of DA that may account for the observed alterations. It is important to note however, that prenatal TURP treatment caused two mechanistically differentiable effects in the offspring. One was the increased response to a single AMPH injection, which may stem from changes in the release of DA in the NAcc [54], therefore is more directly related to increased basal expression of TH and to the increased basal content of DA in the NAcc. In contrast, behavioral plasticity after repeated AMPH administration is a phenomenon that requires long-term neural adaptations that occur in several brain structures, notably the VTA, where the cell bodies of the mesolimbic DA neurons lie [55]–[57], therefore TURP may affect additional cellular and molecular targets. In this regard, iron supplementation to TURP-treated mothers seems to have prevented only some of the effects of TURP that impact the response to acute AMPH injection (i.e. increased NAcc TH expression, but no greater DA and metabolites) as well as those mechanisms underlying the sensitized response after repeated AMPH administration.

Interestingly, iron supplementation alone (i.e. SAL-IRON group) induced several behavioral and biochemical changes in the offspring, which resembled the effects of prenatal inflammation (i.e. enhanced sensitivity to acute and repeated AMPH injection and increased basal levels of DA, DOPAC and HVA in the NAcc). SAL-IRON group also exhibited a greater AMPH-induced stereotypy during the pre-treatment phase (data not shown) and increased basal levels of DOPAC in the dSTR. This surprising finding is most likely due to iron overload in iron sufficient animals [58], although the doses of iron used in our study were chosen to be below those that cause toxic side effects [59], [60]. This is further exacerbated by the fact that parenteral administration of iron bypasses the regulatory mechanisms that control its dietary absorption [26], therefore iron administered through dietary supplementation, which normally does not cause overload [61], may spare the offspring from the undesired effects described in the present study. To the best of our knowledge, there are no studies on the effects of maternal-iron supplementation on the DA function of the adult offspring, however it is normally considered that excess of iron results in toxic effects due to oxidative stress [26]. These findings, although unexpected, nevertheless emphasize the critical importance of this micronutrient to normal development of mesolimbic DA neurons.

Given that maternal circulation constitutes the only source of iron to the developing fetus [38], and that HAMP down-regulates FPN1 [62], which is essential for the transport of iron through the materno-fetal interface [63]–[67], we rationalized that the inflammation-induced reduction in maternal iron will restrict the amounts being transported through the materno-fetal interface. To confirm this, we measured iron levels in the placenta, fetal liver and brain collected from all the treatment groups. As expected, placental iron levels were dramatically reduced, paralleling the maternal supply of this nutrient and completely recovered by iron replenishment in TURP treated dams. The TURP-induced reduction in placental iron content may reflect either the reduced availability of maternal serum iron and/or the demand to sustain the fetal development during the hypoferremia [68].

The changes in iron levels in the placenta were very closely paralleled by the levels of HAMP expression, which may be merely responding to the tissue's fluctuation in iron concentration [69] or conversely, responding to a signal from outside the placenta, and in turn modulating iron export rates from this tissue to the fetal compartment [68]. Interestingly, regulation of HAMP mRNA expression in fetal liver was the reverse of that seen in the placenta. In this tissue, TURP-treatment resulted in induced expression of HAMP mRNA at a time point when TURP-induced maternal circulating cytokines are no longer elevated, suggesting that mediators other than cytokines may be involved in this effect. One likely possibility is that iron flow from the placental stores into the fetus resulted in higher than normal iron levels in the fetal circulation, thus triggering fetal liver HAMP mRNA expression [68]. In fact, fetuses of iron supplemented (SAL-IRON) dams also showed increased fetal liver HAMP mRNA expression, which is presumably due to a direct effect of the supplemented micronutrient [68], as no inflammatory pathways were activated in this condition.

Iron levels in the fetal brains taken from all four treatment groups did not show any alteration, other than a small but significant reduction in TURP-IRON group. This latter observation is rather puzzling and it possibly reflects a lower than normal supply of iron in the TURP-IRON group. The latter effect can be mediated by the increased placental HAMP in the TURP-IRON group, which would lead to iron trapping within this organ, at the expense of the fetal brain, similar to the effects of maternal hepatic HAMP on maternal liver iron content [70]. However, it is important to note that in the present study we measured whole brain iron levels, and that marked regional differences in iron acquisition capabilities of the brain are possible and have indeed been reported [30], [71], [72]. In fact, brain DA regions are known to be enriched in iron [73], [74] and susceptible to systemic variations of this nutrient [75], suggesting that specific effects of inflammation and iron supplementation may be found in the mesolimbic DA neurons, which would then be involved in the induction of the marked effects on DA function observed in the adult offspring. Altogether our data are consistent with a scenario where inflammation-induced maternal hypoferremia leads to placental deficiency in this nutrient. This could be due, at least partly, to a putative increase in iron transport to the fetal compartment that keeps whole-tissue levels of iron normal. In addition, placental iron deficiency may in turn result in altered function of this tissue. For example, dietary iron deficiency leads to increased cytokine expression in the placenta [76] and altered transport of amino acids towards the fetal compartment [77], [78]. This may, along with direct restriction of iron supply in specific brain areas, impact aspects of neuronal development, resulting in the effects observed in the adult offspring.

Hypoferremia has long been recognized as one of the main components of the host's response to infection/inflammation, and the mechanisms involved in the induction of this process are fairly well understood. The strongest evidence to date suggests that the pro-inflammatory cytokine IL-6, which is readily detectable in the circulation of infected individuals regardless of the pathogenic trigger, is the main circulating mediator of hepatic HAMP mRNA expression [19]–[22]. IL-6 has been suggested to be strongly involved in the etiology of the offspring alterations induced by maternal inflammation [79]. We cannot rule out the direct involvement of either other cytokines [45] or other mediators, such as inflammation-induced alteration in zinc homeostasis (hypozincemia) [80]. However, our results strongly indicate that during prenatal inflammation, hypoferremia plays a fundamental role in the developmental effects of this maternal insult on the mesolimbic DA function, and that the effect of IL-6 on the developing fetus may be mediated trough this mechanism.

Our results clearly demonstrate that a localized inflammatory insult during gestation has profound affects on DA function, which may be relevant for schizophrenia, where increased striatal DA is proposed to underlie the so-called positive symptoms of the disorder [81]–[84]. In addition, we showed for the first time that this risk factor for schizophrenia also increased the animal's vulnerability to develop sensitized behavioral response to an AMPH challenge given one week after repeated exposure. Repeated administration of drugs of abuse, including AMPH, has been shown to also result in sensitization to the rewarding effects of these drugs [52]. Thus, our findings further strengthen the link between these two psychiatric disorders, whose co-morbidity has been proposed to stem from DA dysfunction [85]. Finally, we provided new evidence for the involvement of fetal/maternal iron homeostasis in the developmental processes that render the offspring more susceptible to enhanced DA function.

## Materials and Methods

### Animals

Time pregnant primiparous Sprague-Dawley rats (Charles Rivers, QC, Canada) were used in all experiments. On GD 7 or 8 animals were individually housed in a controlled environment at an ambient temperature of 21±1°C on 12:12 h light-dark cycle (lights on at 0800 hours) with free access to food and water. Only male offspring were used, which were housed under the same environmental conditions. Mothers were treated with the inflammatory stimulus at GD 15. This day of pregnancy was used since it has been proposed to be roughly equivalent to the late 1^st^ trimester of human pregnancy [86], which has been significantly linked with the increased risk of developing schizophrenia in the offspring of infected mothers [1]. Importantly, our previous studies in pregnant rats treated with TURP at GD 10, 15 or 18, suggested a window of vulnerability for the adult offspring treated at GD 15 [7].

*Ethics statement.* Experimental procedures were approved by the Animal Care Committee from the Douglas Mental Health University Institute and McGill University pursuant of the Canadian Council of Animal Care (Animal Use Protocol # 4306). All efforts were made to minimize the number of animals used.

### Treatments and experimental protocols

Pregnant dams were handled daily from GD 11 onwards and habituated to a rectal probe to determine core temperature (Physitemp Instruments, NJ, U.S.A.). Some animals (13 dams) received an intraperitoneal (i.p.) injection of 10 mg/kg of the aqueous complex of poly-nuclear iron (III)-hydroxide in sucrose (Venofer, American Reagent, NY, U.S.A.) or an equivalent volume of vehicle (either saline [7 dams] or 30% sucrose [7 dams]) at GD 13 and 14. Iron was administered to the dams from GD 15 until GD 18 at larger doses (i.p., 20 mg/kg). At GD 15 basal core temperature was recorded and a small sample of tail blood was collected to determine basal levels of cytokines and serum iron. Subsequently, a maternal inflammatory response was elicited by injecting intramuscularly with 100 µL of purified TURP (Riedel-deHaën, Sleeze, Germany); control dams received an equivalent volume of saline. All injections were administered between 0900 and 1000 hours. Using a rectal probe core body temperature was measured at 8, 10, 24, 48 and 72 hours after i.m. treatment and tail blood collected at each time point. The animals were then sacrificed at GD 19 (96 hours after the i.m. treatment) with a lethal dose of pentobarbital sodium (i.p., 60 mg/kg) and a final blood sample was collected via cardiac puncture. Maternal liver, placentas, fetal livers and brains were excised and immersed in 2-methylbutane (Fisher Scientific, Hampton, NH, U.S.A.) and chilled with dry ice. Serum cytokine, iron and Tf saturation levels were determined, as well as maternal liver, placenta, fetal liver and brain tissue iron levels.

In a separate experiment, SAL and TURP treated dams (i.m., 7 rats per group) were sacrificed 10–11 hours after i.m. treatment, which represents the peak of fever and cytokine responses [87], and livers collected to measure mRNA expression of molecules involved in iron homeostasis.

To determine the effects of prenatal TURP and iron supplementation on DA function and related behaviors in the adult offspring, pregnant dams (6–12 per group) were treated as described above, with the exception that tail blood collection was not performed. The male offspring from each mother were marked, weighed and cross-fostered with surrogate dams in mixed litters [6], [7]. Offspring were weaned from their foster mothers at postnatal day (P) 22 and used at P 60–62, for either behavioral testing or biochemical analyses. Behavioral sensitization to AMPH was used to gauge midbrain DA function and plasticity in the adult offspring. In addition, protein expression levels of TH were determined by Western blotting from extracts obtained from the VTA, SN, NAcc, dSTR and mPFC. Tissue levels of DA and metabolites (DOPAC and HVA) were determined by High Performance Liquid Chromatography (HPLC) from NAcc, dSTR and mPFC.

### Behavioral testing

We measured locomotor responses to either single or repeated injections of d-amphetamine sulphate salt (AMPH, Sigma-Aldrich, Dorset, UK) in adult male offspring, using our well-established protocol [57]. Briefly, locomotor activity was quantified with an infrared activity-monitoring apparatus for rats (AccuScan Instruments, Columbus, OH, U.S.A.). On day 1 all rats (n = 23–50 per group) were habituated to the boxes for 30 minutes (basal locomotor activity). On day 2 all animals received a saline injection (1 µl/g, i.p.) and were placed back in the boxes for 30 minutes. Immediately after, one half of the animals (saline pre-treatment group, 11–26 per group) received another saline injection, and the other half was injected with AMPH (2 mg/kg, i.p.); locomotor activity was monitored for an additional 90 minutes. On days 3, 4, 5 and 6, animals received an injection of either saline (saline pre-treatment group) or AMPH (2 mg/kg, AMPH pre-treatment group) and their locomotor activity was measured for 90 minutes. Finally, on day 14, a week after termination of saline or AMPH pre-treatment, a test for behavioral sensitization was conducted, where all animals, regardless of the pre-treatment condition, received a single injection of AMPH (1 mg/kg, i.p.). Locomotor activity was monitored for 90 minutes. This lower dose of AMPH for the sensitization test was chosen to avoid stereotypy. All behavioral measurements were performed between 0900 and 1600 hours.

### Serum cytokine and iron determination

All blood samples were allowed to cloth at room temperature for 1 hour, spun at 4000 rpm for 20 min at 4°C to obtain serum and stored at −80°C until used. Maternal serum samples were analyzed for IL-6 and IL-1ra (the main cytokines released into the circulation by TURP treatment [87]) using species specific ELISA (NIBSC, Potters Bar, UK) as described previously [88]. Serum iron (SI) was measured using an iron kit (RANDOX, Mississauga, ON, Canada). In parallel, a direct measure of total iron binding capacity (TIBC) of the serum was determined using a TIBC kit (RANDOX). For both procedures the manufacturer's protocols were followed. Once both measurements were obtained (expressed as µg/dL of serum), serum T) saturation was calculated by expressing the serum iron content as percentage of TIBC (Tf saturation  =  SI/TIBC×100).

Three fetuses per dam were collected, and the results from their individual measurements averaged to obtain one value per mother. For tissue iron content, maternal liver, placenta, fetal liver and brain were homogenized in 1∶10 (w/v) high-purity water. One volume of protein-precipitation solution (1 N HCl and 10% (v/v) trichloroacetic acid in high purity water) was then added to the samples, blank and iron standards and incubated for 1 hour at 95°C. The samples were then allowed to cool at room temperature for 2 min, vortex mixed and centrifuged at 10 000×*g* for 10 min at room temperature [89], [90] and assayed for iron content.

### Quantitative RT-PCR

Total RNA was extracted and reverse transcribed from maternal livers, placenta, fetal liver and brain as described previously [91]. Real-time PCR was performed in duplicate using pre-optimized primer/probe mixture (TaqMan Gene Expression Assays, Applied Biosystems, ON, Canada) and TaqMan universal PCR master mix (Applied Biosystems). The housekeeping gene 18S was used to normalize levels of cDNA expression for each sample. Levels of gene expression were calculated as the X-fold difference from the control groups (SAL-VEH group). The mRNA levels of the following genes was assessed: SOCS3, used as a marker of activity of the JAK/STAT3 signaling pathway activated by IL-6 [92]; HAMP and zip14, a dual iron/zinc importer that has been shown to be induced by TURP and may be involved in the cellular sequestration of iron [40], [93]. The assay ID for each gene is as follows: SOCS3: Rn00585674-s1, HAMP: Rn00584987-m1, zip14 (slc39a14): Rn01468335-g1 and 18S: EUK-18S-rRNA4352930.

### Western blotting and HPLC

We explored biochemical markers of DA neurons, including protein expression of TH and tissue levels of DA and its metabolites, DOPAC and HVA. Brains collected from adult male offspring (P 60–62) by decapitation were immersed in 2-methylbutane (Fisher Scientific, Hampton, NH, U.S.A.) and chilled with dry ice. 300 µm thick cryostat sections were obtained. There were no behavioral differences between the animals born to mothers supplemented with either SAL or sucrose. Thus, to minimize the number of animals used, only SAL-SAL and TURP-SAL groups were included. Bilateral punches from the VTA, SN, NAcc (including core and shell), dSTR and mPFC (including cingulated areas 1 and 2) were excised using our previously described procedures [57] using the Paxinos and Watson rat brain atlas [94].

Western blots were conducted as previously described [57], [87]. Membranes were incubated with antibodies against TH (1:5000, Chemicon, Temicula, CA, U.S.A.) and actin (HRP-coupled, 1∶5000, Santa Cruz Biotechnology, Santa Cruz, CA, U.S.A.). Data are expressed as a ratio of TH over actin optical densities.

HPLC analysis was conducted as we recently reported [95]. Briefly, tissue punches were re-suspended in 100 µL 0.1 m phosphate buffer, pH 7.0, and filtered using 0.45-µm syringe filters. A 10-µL volume of this filtrate was loaded onto a 15-cm C-18 reverse-phase column via manual injection ports (20-µL loop). Dual-channel coulometric III detectors (model 5100A; ESA, Inc., Bedford, MA, U.S.A.) were used to measure the reduction and oxidation currents for DA and DA metabolites (one channel was used for DA and the other for DA metabolites). EZChrom Data Chromatography Data System (Scientific Software, Inc., San Ramon, CA, U.S.A) was used to analyzed and quantify DA, DOPAC and HVA concentrations.

### Data analyses

All data were analyzed as raw values, except for HPLC results, where the data were analyzed as percentage of the average value for the SAL-VEH group, due to large inter-assay variability between two different batches of samples assayed. All data are presented as mean values ± s.e.m. *p* values<0.05 values were deemed significant.

Maternal fever, serum cytokines and iron: data were analyzed using a repeated measures 2-way ANOVA, with time as a within-group variable and pre-natal treatment (SAL vs. TURP) as a between-groups variable.

Maternal liver PCR data: data from the 10–11 hours experiment were analyzed using two-tailed Student's *t*-tests to compare data from the SAL-VEH and TURP-VEH groups.

Maternal serum Tf saturation: data were analyzed using a 3-way ANOVA with prenatal treatment (SAL vs. TURP) and maternal supplementation (VEH vs. IRON) used as between-groups variables in all cases and time point used as a within-group variable. When the interactions of main effects were significant, analysis was followed by performing simple-effects ANOVA and Fisher's LSD post hoc tests.

Tissue iron content, HAMP mRNA levels (96 h time point), western blotting and HPLC: data were analyzed using 1-way ANOVA, with maternal treatment (SAL-VEH vs. TURP-VEH vs. SAL-IRON vs. TURP-IRON) as between-groups variable and furthered with Fisher's LSD post hoc tests when ANOVA was significant.

Offspring's basal and AMPH-induced locomotion: data were analyzed using 3-way ANOVAs, with prenatal treatment (SAL vs. TURP) and maternal supplementation (VEH vs. IRON) as between-groups variables and time as a within-group variable. When interactions were significant, analyses were followed by simple-effects ANOVA and Fisher's LSD post hoc tests.

AMPH sensitization test: data were analyzed using a 3-way ANOVA, with prenatal treatment (SAL vs. TURP), maternal supplementation (VEH vs. IRON) and pre-treatment (saline vs. AMPH) used as a between-groups variables, followed by simple-effects ANOVA and Fisher's LSD post hoc tests when interactions were significant.

## Supporting Information

Figure S1 Inflammatory response remained intact in iron supplemented mothers. (A) Febrile response followed the same kinetics in vehicle and iron supplemented mothers, peaking at 10 h after TURP injection and returning to baseline 24 h later. Basal temperature was not affected by iron supplementation. (B and C) Serum IL-6 and IL-1ra levels followed the same kinetics in the TURP-IRON group compared to the TURP-VEH group.
(4.70 MB TIF)
