# Supplementary figures and images for: Prenatal Inflammation-Induced Hypoferremia Alters Dopamine Function in the Adult Offspring in Rat: Relevance for Schizophrenia
Source: PLoS One. 2010 Jun 4;5(6):e10967. doi: 10.1371/journal.pone.0010967 (PMC2881043; doi:10.1371/journal.pone.0010967)

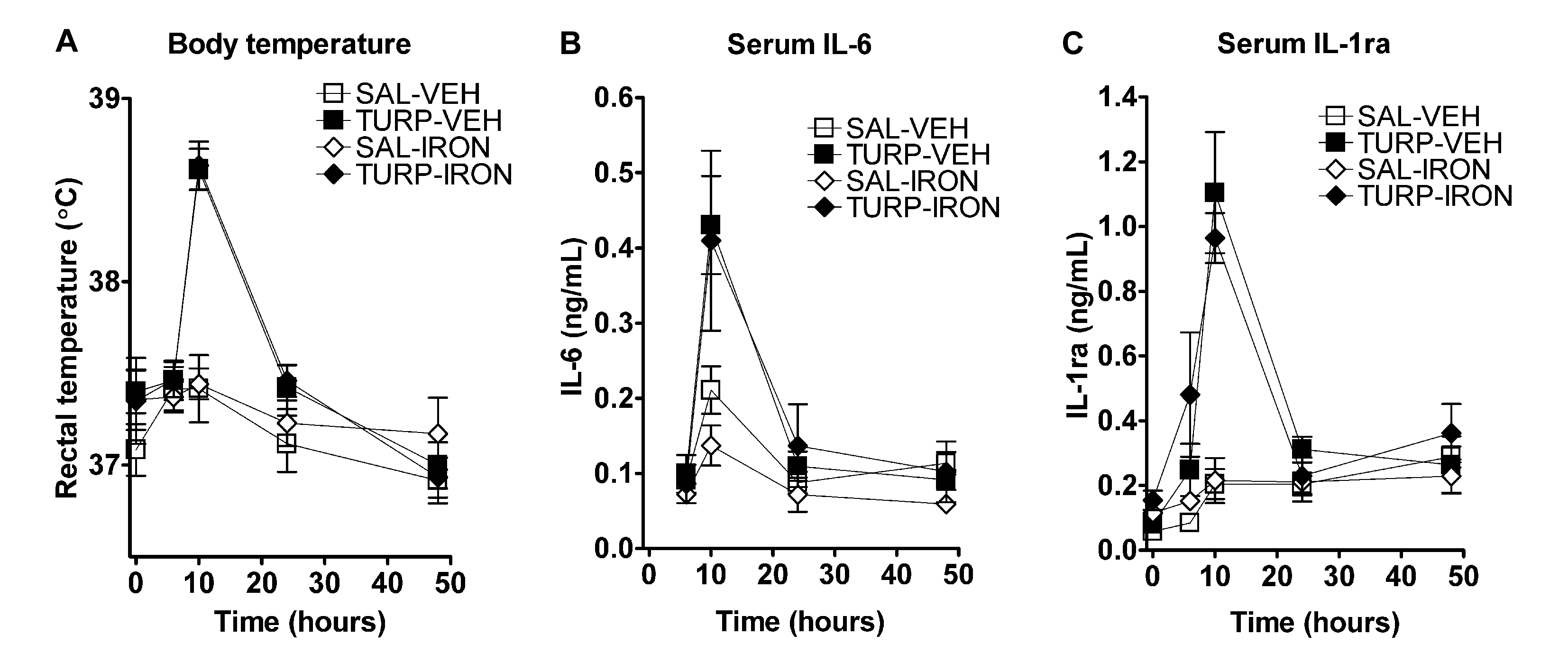

Supplement: Figure S1 — Inflammatory response remained intact in iron supplemented mothers. (A) Febrile response followed the same kinetics in vehicle and iron supplemented mothers, peaking at 10 h after TURP injection and returning to baseline 24 h later. Basal temperature was not affected by iron supplementation. (B and C) Serum IL-6 and IL-1ra levels followed the same kinetics in the TURP-IRON group compared to the TURP-VEH group. (4.70 MB TIF) [file pone.0010967.s001.tif]
